# Security Risk Intelligent Assessment of Power Distribution Internet of Things via Entropy-Weight Method and Cloud Model

**DOI:** 10.3390/s22134663

**Published:** 2022-06-21

**Authors:** Siyuan Cai, Wei Wei, Deng Chen, Jianping Ju, Yanduo Zhang, Wei Liu, Zhaohui Zheng

**Affiliations:** 1 Hubei Province Key Laboratory of Intelligent Robot, Wuhan Institute of Technology, Wuhan 430079, China; csy@stu.wit.edu.cn (S.C.); 15011801@wit.edu.cn (W.W.); zhangyanduo@hotmail.com (Y.Z.); liuwei@wit.edu.cn (W.L.); 2 School of Artificial Intelligence, Hubei Business College, Wuhan 430079, China; jjp@hbc.edu.cn; 3 School of Mathematics and Physics, Wuhan Institute of Technology, Wuhan 430079, China; zhengzhaohui@whu.edu.cn

**Keywords:** power distribution Internet of Things, security risk assessment, evaluation index system, entropy-weight method, cloud model

## Abstract

The current power distribution Internet of Things (PDIoT) lacks security protection terminals and techniques. Network security has a large exposure surface that can be attacked from multiple paths. In addition, there are many network security vulnerabilities and weak security protection capabilities of power distribution Internet of Things terminals. Therefore, it is crucial to conduct a scientific assessment of the security of PDIoT. However, traditional security assessment methods are relatively subjective and ambiguous. To address the problems, we propose to use the entropy-weight method and cloud model theory to assess the security risk of the PDIoT. We first analyze the factors of security risks in PDIoT systems and establish a three-layer PDIoT security evaluation index system, including a perception layer, network layer, and application layer. The index system has three first-level indicators and sixteen second-level indicators. Then, the entropy-weight method is used to optimize the weight of each index. Additionally, the cloud model theory is employed to calculate the affiliation degree and eigenvalue of each evaluation index. Based on a comprehensive analysis of all evaluation indexes, we can achieve the security level of PDIoT. Taking the PDIoT of Meizhou Power Supply Bureau of Guangdong Power Grid as an example for empirical testing, the experimental results show that the evaluation results are consistent with the actual situation, which proves that the proposed method is effective and feasible.

## 1. Introduction

The techniques of the Internet of Things (IoT) have been widely used in electric power distribution networks and form the PDIoT. Different from the traditional power distribution network (PDN), PDIoT has may distinctive characteristics: (1) it has a complex network architecture, (2) master stations are located in the cloud, (3) electric terminal devices are connected by the IoT, and (4) it has a flexible architecture, which can be freely expanded. The above characteristics make the PDIoT more vulnerable than traditional PDN. In recent years, the network security situation has become increasingly severe, and the security events of IoT and industrial control systems [1,2] have increased year by year [3,4]. Through analysis, we found that most of the security problems in PDIoT originated from the following sources: sensors, the network, and terminal devices. Some functional components in the sensors may be attacked to obtain abnormal data and affect system stability. In addition, with functional components as a pluggable expansion module, there may be security risks, permission abuse, and other issues. Network security has a large exposure surface, and a large number of terminals and network interfaces will be deployed to the user side and all levels of system nodes. Malicious attackers can gain physical access to a very large number of points, and these points are difficult to monitor in a comprehensive and timely manner. The terminal devices can attack more paths, such as tablets and other mobile terminals running on various types of third-party developed measurement and control equipment management software, and there may be data leakage, malicious attacks, abuse of privileges, and other abnormal behavior. Therefore, scientific evaluation of the safe and reliable performance of the PDIoT system and a timely grasp of the operation and maintenance status of the distribution network are of great significance to guarantee the security of the PDIoT.

At present, the security assessment of the PDIoT is facing problems of subjectivity and repetition. The traditional reliability assessment method is rule- or model-driven. The main research methods include the fuzzy comprehensive evaluation method, principal component analysis, analytic hierarchy process, etc. For example, Guo et al. [5] proposed a security risk evaluation method for urban power grids based on the fuzzy comprehensive evaluation method, and calculated the security risk level of a city, providing a basis for power grid enterprises to put forward risk control measures in terms of management measures, technical measures, and working standards. However, there is a strong subjectivity in determining the index weights with a complicated calculation process. He et al. [6] applied the principal component analysis method to reduce the dimensions and compress the original variables of power equipment status to obtain the principal component system, and then established a comprehensive evaluation model based on the principal component system to perform a comprehensive and objective evaluation of power equipment status, which has certain practicability. However, the meaning of the integrated evaluation function in this method is unclear when the sign of the factor loadings is positive or negative. Lu et al. [7] designed a state evaluation method of an electric energy metering device by using the analytic hierarchy process and obtained the conclusion of fuzzy evaluation of the operating state of electric energy metering devices. However, it is difficult to conduct consistency tests on the judgment matrix, and the selection of test criteria also lacks a sufficient basis [8].

In response to the above problems, we establish a security evaluation index system for PDIoT based on sensors, networks, and terminal devices, and abstract it into three levels: sensing layer, network layer, and application layer. We record these three levels as primary evaluation indexes, and establish secondary evaluation indexes under each primary index, totaling 16. The entropy power method is introduced to establish the evaluation matrix of PDIoT indexes and carry out the structural entropy calculation, and the cognitive blindness is processed to obtain the weight coefficient ratio of evaluation indexes, which can combine subjective and objective assignment [9]. The cloud model theory is used to study the safety evaluation of the PDIoT system, which can solve the problems of complexity and uncertainty and reveal the inner relationship between randomness and fuzziness [10], which is more consistent with objective facts and higher accuracy of evaluation results than traditional evaluation methods, and makes the evaluation results more intuitive and accurate.

We applied the above method to conduct a security risk assessment on the PDIoT system of the Meizhou Power Supply Bureau of Guangdong Power Grid, and the experiment shows that the security risk level of the PDIoT in this area is “better”, in which the security risk of the network layer is slightly higher, and the security of the sensing layer and the application layer is better. The overall evaluation results are consistent with the facts.

Our main contributions can be summarized as:

- Proposing a novel approach to PDIoT security assessment, combining subjective and objective assignment of evaluation indicators, while enabling the interconversion between qualitative and quantitative evaluation indicators, as well as making the evaluation results more intuitive and accurate.
- Constructing a new security evaluation index system for PDIoT and scoring criteria.
- Putting forward improvement suggestions for modules of potential security risks for the PDIoT.

The rest of this paper is organized as follows. In Section 2, we construct the evaluation index system scientifically and systematically, and set the scoring criteria and principles according to the characteristics of PDIoT. In Section 3, we introduce the entropy-weight method and calculate the weight of each index based on the entropy-weight method. In Section 4, we introduce the cloud model theory, build a comprehensive cloud model of the PDIoT, and use the PDIoT system of Meizhou Power Supply Bureau of Guangdong Power Grid as an example to carry out an empirical test to determine the security level of the PDIoT system in the region and provide the corresponding analysis of the evaluation results. The main conclusions of this paper are presented in Section 5.

## 2. Construction of Evaluation Index System

### 2.1. Construction of Security Evaluation Index

**Figure 1:**
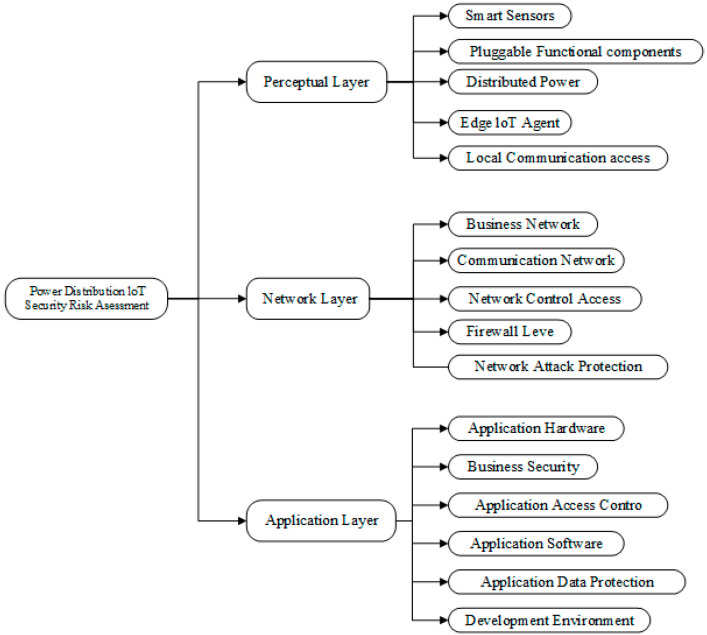
Evaluation index of three-layer architecture of PDIoT system.

The establishment of the evaluation index system of PDIoT should conform to the principles of systemic and scientific evaluation and be operable, and the evaluation indexes should be independent of each other [11,12]. According to the fact that the PDIoT has a similar architecture to other IoT applications and is basically the same in terms of technology and functional level, and the security flaws of PDIoT mainly come from three aspects: sensors, network, and terminal devices, we abstracted it into three levels: perception layer, network layer, and application layer [13]. We recorded these three levels as the first-level evaluation index, referring to the relevant standards of the PDIoT, and established a second-level evaluation index under each first-level index, with a total of 16 indicators, as shown in Figure 1.

### 2.2. Scoring Criteria and Principles

Based on the characteristics of PDIoT and combined with the security evaluation theory, we divided the security evaluation level of the PDIoT system and the rating of each evaluation index into five levels: “excellent”, “superior”, “moderate”, “poor”, and “awful”. Indicators were unified using a 10-point scale, that is, all evaluation indicators were assessed in the range of [0, 10], with higher scores indicating higher security. According to the relevant industry standards, operating procedures, and expert recommendations, we divided the evaluated values into five intervals, namely: [0, 3), [3, 5), [5, 7), [7, 9), and [9, 10], and the corresponding five levels are “awful”, “poor”, “moderate”, “superior”, and “excellent”.

## 3. Determination of Evaluation Index Weights

### 3.1. Entropy-Weight Method

In the whole evaluation system, different weights need to be assigned to each indicator due to the varying importance of each [14]. We used the entropy-weighting method to determine the weights of each evaluation index, which can balance the subjectivity and objectivity of the weight calculation and combine qualitative analysis with quantitative analysis.

(1) Expert opinion collection. In this study, we conducted a questionnaire survey with five experts, including technical leaders and senior engineers of a power supply section, etc. The five experts independently provided their opinions on the importance of each evaluation index set based on their professional knowledge and practical experience in an anonymous manner. The importance ranking is shown in Table 1. The higher the ranking, the higher the importance.

(2) Blindness analysis of typical ranking [15]. We used entropy theory to calculate the entropy values of the three first-level indicators: perception layer, network layer, and application layer, to reduce the inconsistency and uncertainty in the ranking of different indicator systems by various experts. The specific method was as follows: the set of indicators corresponding to each consulting expert is denoted as$\;U=\{u_{1},u_{2},\ldots ,u_{n}\}$, and the ranking array corresponding to U is denoted as $\left(a_{i1},a_{i2},\ldots ,a_{in}\right)$, so as to obtain the ranking matrix of each indicator,$\;A={\left(a_{ij}\right)}_{k\times n}$.


$$A=\left[\begin{array}{cccc} a_{11} & a_{12} & \cdots & a_{1n}\\ a_{21} & a_{22} & \ldots & a_{2n}\\ \ldots & \ldots & \ldots & \ldots \\ a_{k1} & a_{k2} & a_{k3} & a_{kn} \end{array}\right]$$

Calculating the affiliation degree of each expert index: Through qualitative transformation of the above expert opinion according to the information entropy function $\chi \left(l\right)=-\lambda p_{n}\left(l\right)\ln\left(p_{n}\left(l\right)\right)$, the affiliation degree of each expert index is calculated:

(1) $$p_{n}\left(l\right)=\frac{m-l}{m-1},\;\lambda =\frac{1}{\ln\left(m-1\right)}$$

where l is the number of the important ranking given by experts according to the first-level indicators, k is the number of experts involved in the consulting survey, here take k=3, and n is the number of evaluation indicators: as there are three first-level indicators, take n=3. $a_{ij}\;$is the evaluation of the ith expert on the jth indicator, $u_{j}$, which can also be expressed as the number of the ranking of a certain expert on an indicator. The value range of $a_{ij}\;$is {1, 2,..., *j*}, where j represents the maximum ordinal number, here take j=3. m is the number of transformed parameters, take m=j+2, that is m=5. Make:

(2) χ(l)(m−l)(m−1)−1=u(l) 

where u(l) is a function defined as [0, 1]. Bringing each A in matrix$\;l=a_{ij}$ into the above equation, for the quantitative transformation of $a_{ij}$, the corresponding affiliation function $a_{ij}\;$of$\;u\left(a_{ij}\right)$ is rewritten as:

(3) $$u\left(a_{ij}\right)=\frac{\ln\left(m-a_{ij}\right)}{\ln\left(m-1\right)}$$

The affiliation matrix$\;B={\left(b_{ij}\right)}_{k\times n}\;$is obtained by setting $b_{ij}=u\left(a_{ij}\right)$. Approximating that the experts involved in the research have the same “right to speak”, i.e., the ranking of each evaluation index, $u_{j},\;$is equally important, the average affiliation of the ranking number of five experts in the evaluation index, $u_{j},\;$is called the average recognition degree, which is denoted as $b_{j}$, so that:

(4) $$\;b_{j}=\left(b_{1j}+b_{2j}+\ldots +b_{nj}\right)/k$$

Let the uncertainty generated by the expert’s perception of the PDIoT assessment factor $u_{j}$ be the awareness blindness, denoted as $Q_{j}$, such that:

(5) $$\;Q_{j}=\sqrt{\frac{1}{k}\overset{k}{\underset{i=1}{\sum }}{\left(b_{ij}-b_{j}\right)}^{2}}$$

Then, the overall awareness degree $\left(x_{j}\right)$ of the *k* experts is:

(6) $$\;x_{j}=b_{j}\left(1-Q_{j}\right),x_{j}>0$$

(3) Normalization is carried out to obtain the indicator, $u_{j}$, weights, which are normalized to $x_{j}=b_{j}\left(1-Q_{j}\right)$. Let:

(7) $$v_{j}=x_{j}/\overset{n}{\underset{i=1}{\sum }}x_{i}$$

### 3.2. Results of Weight Calculation

According to the calculation process of the above steps, the weights, $v_{j}$, of the primary indicators can be obtained, and the above method was also used to calculate the weights of the secondary indicators under each primary indicator. The calculation results are shown in Table 2.

## 4. Cloud Theory and the Construction of the Cloud Evaluation Model

### 4.1. Definition of Cloud Model

The cloud model is a cognitive computing model that realizes the conversion between qualitative and quantitative representation on the basis of the combination of probability theory and fuzzy mathematics theory. It can reflect the internal relationship between fuzziness and randomness and establish the mapping between qualitative and quantitative data. In recent years, this method has been widely used in various fields, such as data mining [16], algorithm improvement [17], system measurement [18], and decision support [19].

The cloud model represents its overall characteristics through three parameters: expectation, Ex, entropy, En, and super-entropy, He. Ex represents the expectation of the distribution of cloud drops in the domain space, which is the most typical sample of this concept of quantification, a point representing the qualitative concept [20]. En reveals the association between vagueness and randomness, and is used to measure the vagueness and probability of the qualitative concept. He is the uncertainty measure of entropy, i.e., the entropy reflects the cohesiveness of the uncertainty of all points representing the linguistic value in the number field space, i.e., the cohesiveness of the cloud drops [21].

### 4.2. Cloud Model Algorithm

The cloud generator is a specific method to realize the cloud model, including forward and reverse cloud generators. Among them, the forward cloud generator generates a cloud map through the characteristic numbers Ex,En,He of the cloud, which can reflect the process from a qualitative concept to a quantitative expression [22]. In this paper, the three digital characteristics of each evaluation index of PDIoT were calculated, and then the standard cloud map and comprehensive evaluation cloud map were generated through the cloud forward generator to evaluate the risk level of PDIoT. The generation algorithm of the cloud graph in the cloud model is the forward cloud generator. The forward cloud generator is an algorithm that can convert qualitative concepts into quantitative values. It was used to generate cloud droplets in this paper. The algorithm of the forward cloud generator is as shown below, Algorithm 1.

**Algorithm 1** Forward cloud generator *Input*: {Ex,En,He};*Output*: $\left\{Drop\left(x_{i},u_{i}\right),i=1,2,\ldots ,n\right\}$
Generate normal random numbers $En_{i}{}^{\prime }\sim N$($En,He^{2}$);Generate normal random numbers $x_{i}{}^{\prime }\sim N$($En,En_{i}{}^{\prime }$);Find the cloud drops $u\left(x_{i}\right)=\exp\left(-\frac{{\left(x_{i}-Ex\right)}^{2}}{2{\left(En_{i}{}^{\prime }\right)}^{2}}\right)$;$u\left(x_{i}\right)$ is the degree of determination and $x_{i}$ is 1 cloud drop in the number field;Repeat steps 1–4 until N cloud drops are generated. Let the left boundary of each rating interval of the evaluation index be $I_{min}$ and the right boundary be $I_{max}$ Cloud parameters (Ex,En,He) are determined by $I_{min}\;$ and $I_{max}$:


(8)
$$\{\begin{array}{c} \begin{array}{c} Ex=\left(\;I_{max}+I_{min}\right)/2\\ En=\left(\;I_{max}-I_{min}\right)/6\\ He=k \end{array} \end{array}$$


where Ex is the sample cloud expectation, En is the cloud entropy value, He is the cloud super-entropy, $En_{i}{}^{\prime }$ is the cloud entropy value of the index after the next iteration, and *k* reflects the randomness and linguistic fuzziness of each evaluation index and is a constant and should not be too large; here, *k* is 0.02.


### 4.3. Determination of Cloud Model Eigenvalues

**Figure 2:**
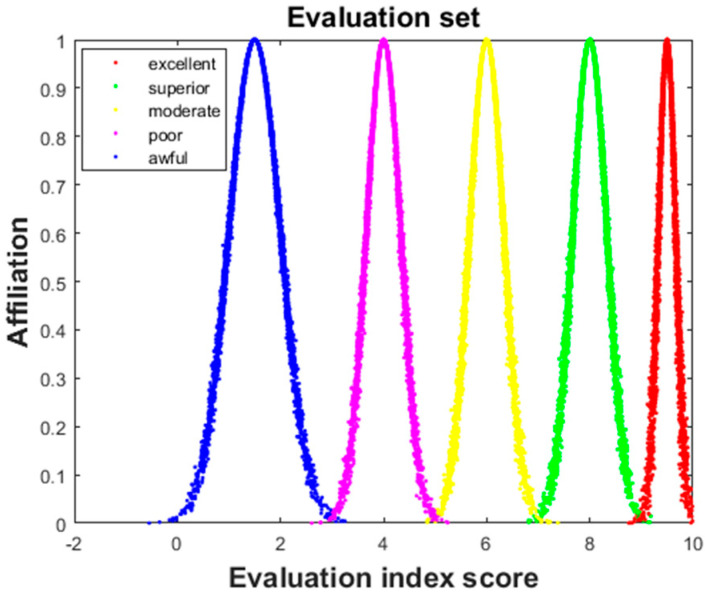
Cloud model with different security levels. Different color point sets represent different security levels: the red represents “excellent”, the green represents “superior”, the yellow represents “moderate”, the purple represents “poor”, and the blue represents “awful”.

Based on the principle of the forward cloud generator, the cloud eigenvalues Ex, En, and He corresponding to different security levels were calculated according to Equation (8), and the results are shown in Table 3. The cloud model diagrams corresponding to different security levels can be calculated according to the calculation results in Table 3 and were drawn with Matlab software (see Figure 2).

### 4.4. Security Evaluation Process

We analyzed the case of the PDIoT system of Meizhou Power Supply Bureau of Guangdong Power Grid. The safety evaluation of this PDIoT system was carried out according to the safety evaluation index system constructed in Figure 1, and experienced engineers of the company were invited to participate in the survey, including two deputy chief engineers and two engineers of the technical section of this power supply section, as well as one deputy chief engineer of each of the other two power supply sections and two engineers of each of the technical sections of the other two power supply sections, for a total of ten participants.

According to the actual situation, the evaluation indexes were scored with reference to the corresponding scoring rules. We considered that although the deputy chief engineer has the advantage in terms of knowledge level and work experience and has more authority, the engineers of the grassroots section have a deeper understanding of the site situation and can compensate for the relative lack of knowledge level and working experience to a certain extent. Therefore, the 10 scores were directly averaged to obtain the final average score. The average scores of each evaluation index are shown in Table 4.

#### 4.4.1. Determination of Integrated Cloud Parameters for PDIoT

Suppose that there are *n* identical types of language concepts, $B_{1}$, $B_{2}$,...,$\;B_{n}$, and $B_{1}\in \left(Ex_{1},En_{1},He_{1}\right)$, $B_{2}\in \left(Ex_{2},En_{2},He_{2}\right)$,...,$\;B_{n}\in \left(Ex_{n},En_{n},He_{n}\right)$, whose weights are $v_{1}$, $v_{2}$,... $v_{n}$ in order; then, these *n* language concepts can form a composite cloud of the same type, and the eigenvalue, B∈(Ex,En,He), of this composite cloud [23] can be calculated as:

(9) $$\{\begin{array}{c} Ex=\overset{n}{\underset{i=1}{\sum }}Ex_{i}En_{n}v_{i}/\overset{n}{\underset{i=1}{\sum }}En_{n}v_{i}\\ En=\overset{n}{\underset{i=1}{\sum }}En_{n}v_{i}\\ He=\overset{n}{\underset{i=1}{\sum }}He_{i}En_{n}v_{i}/\overset{n}{\underset{i=1}{\sum }}En_{n}v_{i} \end{array}\;$$

**Figure 3:**
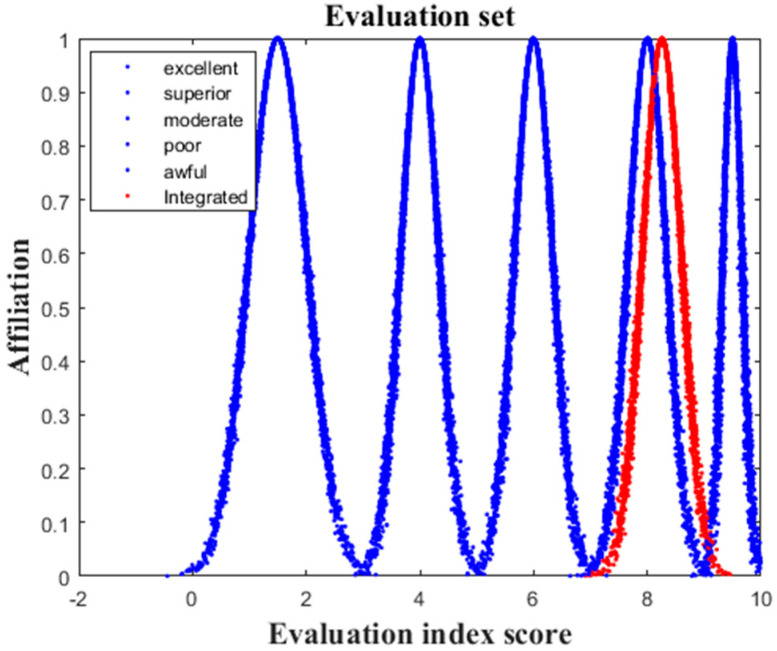
Integrated cloud model. The figure compares the generated integrated cloud model diagram with the standard cloud model diagram to judge the security level of the integrated cloud model. The red represents the integrated cloud model, and the blue represents the standard cloud model.

The resulting weights calculated by the entropy-weight method are shown in Table 4, and according to Equation (8), the eigenvalues of the integrated cloud of the evaluation system were calculated as Ex=8.257, En=0.342, and He=0.02. The integrated cloud model was plotted using Matlab (see Figure 3) and compared with the cloud model images of each security level.

#### 4.4.2. Determination of the Affiliation Degree of Each Level

According to the algorithm of the forward cloud generator, the affiliation degree of each evaluation index score belonging to a certain safety evaluation level was calculated by Equation (8). The process of determining the affiliation degree is illustrated by taking the case of the secondary indicator smart sensor A_11_ under the primary sensor factor A_1_. From Equation (8), the affiliation degree of the index corresponding to the five security levels is $u_{1}=0$,$\;u_{2}=0.793$, $u_{3}=0$,$\;u_{4}=0$, and$\;u_{5}=0$. According to the principle of maximum affiliation, employee culture A_1_ has the highest degree of affiliation for $u_{2}$, so the security level is “superior”. Similarly, other indicators can be determined with the corresponding affiliation of their safety evaluation level.

According to the subordination degree of each evaluation index, the comprehensive subordination degree, *K,* can be calculated from Equation (10), and according to the maximum comprehensive subordination degree, the safety evaluation level of the power distribution Internet of Things system can be judged:

(10) $$K=\overset{n}{\underset{i=0}{\sum }}u_{i}\omega_{i}$$

where$\;\omega_{i}$ denotes the weight of each evaluation index, and $u_{i}$ is the affiliation degree of each evaluation index.

According to the characteristic parameters Ex=8.257, En=0.342, and He=0.02 of the integrated cloud, the affiliation degrees, $u_{1}=0.083$, $u_{2}=0.405$,$\;u_{3}=0$, $u_{4}=0$, and $u_{5}=0$, of the integrated cloud could be obtained, and the integrated affiliation degrees were calculated as 0.083 and 0.405, respectively, according to Equation (8). It can be concluded that the evaluation level of PDIoT in this area was between “excellent” and “superior”, with a preference for “superior”. From Figure 3, we can see that the cloud droplets were dense, the evaluation results were stable, and the evaluation results were consistent with the actual situation of the region, indicating that the evaluation method proposed in this paper is effective and feasible.

#### 4.4.3. Comparative Analysis

The method of this paper is compared with entropy fuzzy set theory, mainly comparing the differences of the evaluation methods, as shown below.

The entropy-weight fuzzy set theory evaluation method determined in [24] was used to evaluate the security of the PDIoT system. The calculated comprehensive risk value was 0.1413, which was substituted into the entropy-weight fuzzy set theory evaluation table, and the evaluation result was “superior”, as shown in Table 5. The results obtained by this method are consistent with those in this work.

Through the comparison of the two evaluation methods, it can be found that the evaluation results obtained by using the cloud model were more intuitive and persuasive. Since the result of the cloud model is C=(Ex,En,He), it contains three values of the cloud model. Not only will the evaluation results (expected values) be displayed visually, but also the entropy (width) and super-entropy (thickness) of the cloud model, which makes it clear and persuasive.

#### 4.4.4. Analysis of Evaluation Results

We can see from the integrated cloud model in Figure 3 that the evaluation result was between “excellent” and “superior”, with a preference for “superior”, and the comprehensive affiliation degrees calculated according to Equation (8) were 0.083 and 0.405. The scores of each index show that the scores of perception layer A_1_ and application layer A_3_ were generally high, which means that most of the index parameters of the PDIoT system were in good condition, the quality of the staff met the requirements, and the operating environment had slight adverse effects but did not cause danger. Therefore, there is overall a low probability of safety accidents. The scores of indicators under network layer A_2_ were generally low, indicating that the security management of the PDIoT system network needs to be strengthened, and the results were consistent with the internal security inspection results of the PDIoT system. During the internal security inspection, it was found that the network firewall was weak and was subject to network attacks from time to time, and the communication network and business network were not sufficiently secure, which could also be reflected in this evaluation system.

In response to the above problems, we have developed the following measures. First, network security management should be strengthened, firewall protection should be enhanced, network supervision should be strengthened, and access rights to both the external and internal networks should be strictly managed. Secondly, the security management system should be continuously revised and improved, the implementation and effective implementation of the system should be guaranteed, and the supervision of the grassroots maintenance personnel should be strengthened to ensure the mastery of the maintenance site situation.

Therefore, by using the cloud model theory, the evaluation of the PDIoT system can be accurately and visually carried out, the security status of the PDIoT system can be grasped, and the weak links can be found in the operation and maintenance of the PDIoT system.

## 5. Conclusions

In this paper, we built a safety evaluation index system containing three first-level indicators and sixteen second-level indicators for the characteristics of PDIoT, combined with the actual site, relevant operation procedures, and management documents. Then, based on the entropy-weight method, we performed an objective evaluation of the evaluation index weight of the PDIoT, and used the entropy theory to objectively correct the evaluation differences of different experts. We introduced the cloud model into the security evaluation of PDIoT to solve the randomness and fuzziness between the security level of PDIoT and different indicators. To verify the feasibility of the method, we analyzed the case of the PDIoT system of Meizhou Power Supply Bureau of Guangdong Power Grid. The evaluation results showed that the characteristic parameters of the integrated cloud of the PDIoT system in this area were Ex=8.257, En=0.342, and He=0.02, and the comprehensive membership degrees were $u_{1}=0.083$ and $u_{2}=0.405$, indicating that the security level in this area was in a “superior” security state, which is consistent with the actual situation of this area, which proved that the evaluation method proposed in this paper is effective and feasible. Then, we compared and analyzed the security evaluation method used in this paper with the entropy-weight fuzzy set method, and found that the evaluation results of the two methods were similar. However, the evaluation results of the cloud model were more intuitive and persuasive. Finally, according to the evaluation results, we put forward some reasonable suggestions for the PDIoT system in this area to reduce the possible security risks. In the future, the deep learning-based technique [25,26,27] will be examined to improve the performance of PDIoT.

## Figures and Tables

**Table 1:** Expert ranking table of importance of first-level indicators.

Experts	A_1_	A_2_	A_3_
Expert 1	1	3	2
Expert 2	1	2	3
Expert 3	2	3	1
Expert 4	1	3	2
Expert 5	2	1	3

**Table 2:** Weight of PDIoT three-level security evaluation index calculated according to the entropy-weight method.

Tier 1 Indicators	Secondary Indicators
Perceptual Layer A_1_ (0.378)	Smart sensors A_11_ (0.262)
	Pluggable functional components A_12_ (0.250)
	Distributed power A_13_ (0.133)
	Edge IoT agent A_14_ (0.173)
	Local communication access A_15_ (0.182)
Network Layer A_2_ (0.214)	Communication network A_21_ (0.223)
	Business network A_22_ (0.244)
	Firewall level A_23_ (0.161)
	Network control access A_24_ (0.262)
	Network attack protection A_25_ (0.129)
Application Layer A_3_ (0.399)	Application software A_31_ (0.124)
	Application hardware A_32_ (0.109)
	Development environment A_33_ (0.140)
	Business security A_34_ (0.198)
	Application access control A_35_ (0.218)
	Application data protection A_36_ (0.211)

**Table 3:** Cloud feature values for different security levels, including three characteristic values: Ex, En, and He.

Security Levels	*E*x	*E*n	*He*
excellent	9.5	0.167	0.02
superior	8.0	0.332	0.02
moderate	6.0	0.332	0.02
poor	4.0	0.332	0.02
awful	1.5	0.332	0.02

**Table 4:** Average score of each indicator.

Index	Average Score	Index	Average Score
A_11_	8.3	A_24_	7.8
A_12_	8.2	A_25_	5.5
A_13_	7.5	A_31_	8.7
A_14_	6.5	A_32_	9.0
A_15_	7.7	A_33_	8.2
A_21_	7.2	A_34_	7.4
A_22_	6.4	A_35_	8.8
A_23_	7.2	A_36_	7.2

**Table 5:** Security risk level based on entropy-weight fuzzy set theory.

Security Levels	Risk Value
excellent	0~0.2
superior	0.2~0.4
moderate	0.4~0.6
poor	0.6~0.8
awful	0.8~1

## Data Availability

Not applicable.

## Abbreviations

The following abbreviations are used in this manuscript:

IoT	Internet of Things
PDIoT	power distribution Internet of Things
PDN	Power distribution network
